# Fluticasone Propionate Liposomes for Pulmonary Delivery

**Published:** 2009

**Authors:** N. M. Nirale, R. D. Vidhate, M. S. Nagarsenker

**Affiliations:** Department of Pharmaceutics, Bombay College of Pharmacy, Kalina, Santacruz (East), Mumbai-400 098, India

**Keywords:** Dry powder inhaler, fluticasone propionate, antistatic agents

## Abstract

The objective of the present study was to entrap fluticasone propionate in liposomes and study *in vitro* lung deposition of both liposomal dispersion and dry powder inhalation using twin stage impinger and Anderson cascade impactor. Liposomes were prepared by lipid film hydration method and characterized for size, shape, morphology, entrapment efficiency and *in vitro* lung deposition. The spray dried liposomes were further characterized for various physicochemical properties such as physical appearance, density, flow properties, drug content and *in vitro* pulmonary deposition. Fine particle fraction was also determined. Liposomal dispersion of fluticasone propionate was successfully prepared with more than 90% entrapment. Spray dried liposomes had mean size of 3-4 μ and a fine powder fraction of 9-10 %. Inclusion of antistatic agents such as leucine and magnesium stearate did not improve the aerosolisation behaviour of dry inhalation powder in this study.

Pulmonary delivery of liposomes has been explored as an alternative to administration of drug agents used in pulmonary disorders. Liposomes offer protection against drug metabolism in the pulmonary tissues. Use of liposomes achieves sustained or prolonged release of drugs in lungs[1,2]. However, use of the system is hampered by long-term instability problems as liposomal dispersions may undergo physicochemical changes resulting in leakage of the encapsulated drug[1]. The dry powder inhalation (DPI) formulation can overcome instability problems of liposome dispersions, offer better stability; ease of administration and patient compliance. Fluticasone propionate (FP) a glucocorticoid administered as DPI formulation, an effective and widely used antiinflammatory agent for treatment of patients with asthma, allergic rhinitis and COPD was selected as a model drug. The objective of the present study was to entrap FP in liposomes and study *in vitro* lung deposition of both liposomal dispersion and DPI using Twin Stage Impinger (TSI) and Anderson Cascade Impactor (ACI).

## MATERIALS AND METHODS

Phospholipids were obtained as gifts from Nattermann Phospholipid GmBH, Germany. Cholesterol (CH) was purchased from Qualigen fine chemicals, Mumbai, India. FP was obtained as a gift sample from Cipla, Mumbai. Lactose was obtained as gift sample from Friesland foods Domo, Netherlands. All other chemicals and solvents were of AR grade and were used without further purification. Freshly prepared distilled water was used throughout the study.

Liposomes were prepared by lipid film hydration method and characterized for its size, shape, morphology, entrapment efficiency and *in vitro* lung deposition by TSI. Two factors (phospholipid composition and FP concentration), two level experimental design was made use of to optimise liposome size and drug entrapment. Liposomal dispersions were spray dried using mini lab spray dryer using lactose as protectant (DL1), magnesium stearate (DL2) and leucine (DL3) as antiadherents to overcome the deagglomeration of spray dried liposomes. The spray dried liposome powder was suitably diluted with pulmonary grade coarser lactose with objective to improve its flow properties and *in vitro* lung deposition. The spray dried liposomes were characterized for various physicochemical properties such as physical appearance, density, flow properties, DSC, XRD analysis, SEM, drug content and *in vitro* pulmonary deposition by ACI. Fine particle fraction (FPF) was determined by ACI using Rotahaler®.

## RESULTS AND DISCUSSION

**Fig. 1:**
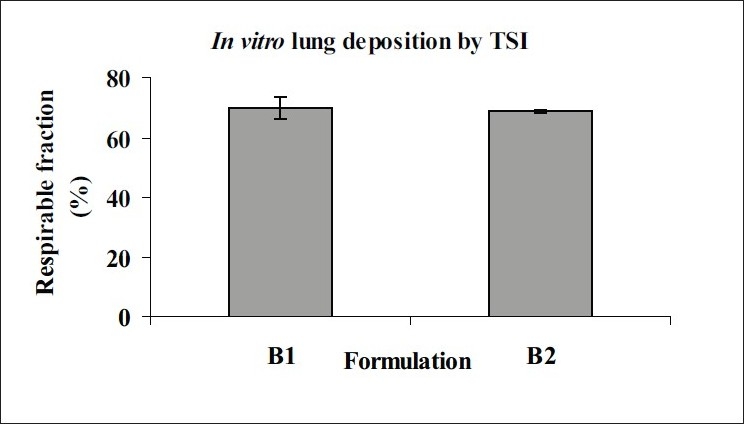
*In vitro* lung deposition of liposomal dispersion B1) Liposomal dispersion with 25 % cholesterol and B2) Liposomal dispersion with 50% cholesterol

**Fig. 2:**
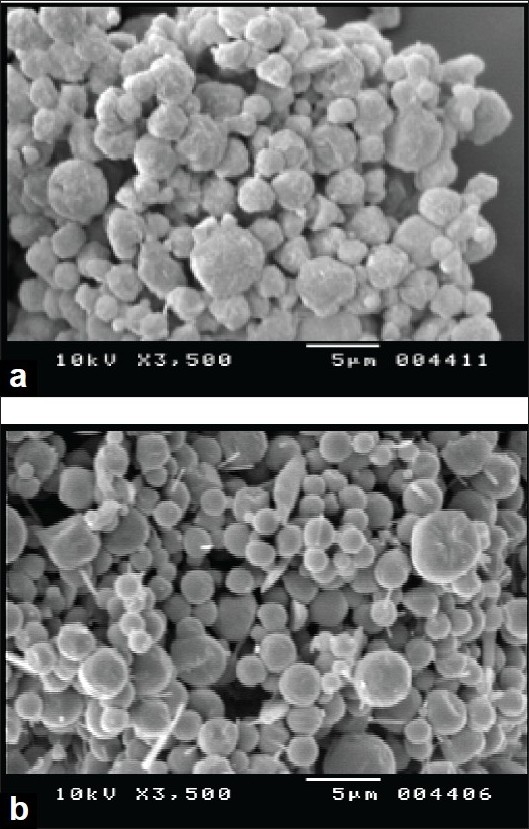
SEM images of spray dried liposomes a) Spray dried liposomal dispersion with leucine (DL3) b) Spray dried liposomal dispersion with magnesium stearate (DL2).

Mean vesicle size in liposomal dispersions was 700-800 nm with polydispersity index of 0.1-0.3 and EE was 95-98%. One or more lamellae were visible in TEM images of blank and FP loaded liposomes. TSI study of FP loaded liposome revealed respirable fraction up to 60-65% (fig. 1). Drug leakage was not significant when products were stored 4° and 25°/60% RH for three months, but particle size increased significantly. The yield of spray drying of liposomal dispersion was in the range of 45-50% and drug content of spray dried liposomes was 98-100%. The DSC and XRD data of spray dried liposomal dispersion showed that FP is amorphised and/or is dispersed in molecular level in the bilayers of liposomes. The SEM images showed spherical morphology of spray dried particles (fig. 2). FPF of spray dried liposomes was about 9-10% (Table 1).

**TABLE 1:** *IN VITRO* LUNG DEPOSITON STUDY BY ACI

Batches	MMAD* (μm)	GSD#	Emitted dose (μg)	Fine Particle Dose (μg)	Fine particulate fraction % (±SD)
DL 1	4.1	1.6	197.60	20.29	10.27
DL 2	3.9	1.5	189.26	18.45	9.75
DL 3	4.0	1.6	187.86	17.25	9.52

* MMAD= mass median aerodynamic diameter

# GSD = geometric standard deviation, DL1 is the spray dried liposomal dispersion with lactose as protectant, DL2 is the spray dried liposomal dispersion with magnesium striate and DL3 is the spray dried liposomal dispersion with leucine.

In conclusion, liposomal dispersion of FP was successfully prepared with more than 90% entrapment. Spray dried liposomes had mean size of 3-4 μ and FPF of 9-10%. Inclusion of antistatic agents such as leucine and magnesium stearate did not improve the aerosolisation behaviour of DPIs in this study. Further studies are warranted in order to improve the FPF.
